# Feasibility of Including Hands-On Microteaching in the Medical Faculty Development Program in India: A Mixed-Methods Study

**DOI:** 10.7759/cureus.40470

**Published:** 2023-06-15

**Authors:** Rekha Dutt, Nihar R Mishra, Ritesh Singh, Sanjay K Patel, Rinchen Dem Dukpa, Soniya C, Atanu K Dutta

**Affiliations:** 1 Community Medicine and Family Medicine, All India Institute of Medical Sciences, Kalyani, Kalyani, IND; 2 Paediatrics, All India Institute of Medical Sciences, Kalyani, Kalyani, IND; 3 Physiology, All India Institute of Medical Sciences, Kalyani, Kalyani, IND; 4 Nursing, All India Institute of Medical Sciences, Kalyani, Kalyani, IND; 5 Biochemistry, All India Institute of Medical Sciences, Kalyani, Kalyani, IND

**Keywords:** kirkpatrick levels, teaching skills, faculty development program, medical education, microteaching

## Abstract

Background

Medical faculty development programs (FDPs) often lack hands-on training in teaching skills. Microteaching, a short, focused teaching practice, could be a feasible way to bridge this gap. This study aimed to explore the feasibility of including hands-on microteaching in a medical FDP in India.

Methodology

This mixed-methods study involved fresh medical faculty members who never attended FDP on the basics of medical education technologies, stakeholders, and students from a recently established autonomous medical institute of national importance in India. Participants completed a pre-test survey on their teaching skills and received a hands-on microteaching session during an FDP. After the session, participants completed a post-test survey and provided feedback on the feasibility and acceptability of hands-on microteaching in the program. Program evaluation was done by application of Kirkpatrick’s Model levels one, two, and three.

Results

According to the Kirkpatrick Model of Evaluation level one (Reaction), the participants reported improved teaching skills and greater confidence in their ability to teach after the microteaching session. They also reported that hands-on microteaching was an effective way to learn teaching skills and receive feedback. Stakeholders opined that microteaching is a very effective tool for improving teaching skills and should be a part of FDP. Evaluation at level two (Learning) shows that there was a significant improvement in the mean score of post-tests. As per level three (Behaviour Change) evaluation, the majority of the students informed that there is observable improvement in the effectiveness of teaching of faculties in the past two months, i.e., since the participation of faculty in hands-on microteaching in FDP.

Conclusions

Hands-on microteaching could be a feasible and effective way to enhance the teaching skills of medical faculty members in India. The study findings suggest that including hands-on microteaching in FDPs could help bridge the gap between theoretical knowledge and practical teaching skills.

## Introduction

Medical education is an ever-evolving field, with new technologies and teaching methods constantly emerging. The quality of medical education is critical to society’s overall health, and one way to ensure high-quality education is through effective faculty development programs (FDPs) [1,2]. Medical FDPs are designed to enhance the skills and knowledge of medical educators and to improve the quality of medical education. Microteaching has been widely used in various education programs that allow instructors to practice their teaching skills in a controlled environment with feedback from their peers and mentors and is an effective way to improve teaching skills [3,4]. Microteaching is a scaled-down version of a teaching scenario that requires less time and a smaller audience, entails less content, which might be covered within a short time of five to seven minutes, and the need to put to use fewer skills and get feedback from their peers and practice their skills as and when required [4].

There is a growing recognition of the importance of incorporating hands-on teaching methods, including microteaching, to improve the quality of medical education [5]. In India, the medical faculty did not get systematic training in teaching skills during their undergraduate or postgraduate period. They learned these skills by observing their teachers [6]. Erstwhile Medical Council of India initiated the training of in-service medical faculty through the FDP and a session on microteaching existed briefly in its schedule of Basic Course Workshop on Medical Education Technologies. This topic is also not found in the three-day FDP schedule recommended by the National Medical Commission (NMC) [7].

Most medical institutes, not under the purview of NMC, conduct FDPs with somewhat varied plans. The Medical Education Unit (MEU) of All India Institute of Medical Sciences (AIIMS), Kalyani, West Bengal, conducted two FDPs including hands-on microteaching sessions by the participants. The data were analyzed to observe the effect of microteaching on the teaching skills of medical faculty and the feasibility to include it in routine FDPs. The findings were presented as a poster at the National Conference of Health Professional Education 2022 held in Dehradun, India on October 31, 2022.

## Materials and methods

Study setting

This study was conducted at the AIIMS Kalyani, West Bengal, 50 km away from the city of Kolkata. The institute is one of the 22 AIIMS newly established under Pradhan Mantri Swasthya Suraksha Yojana with the aim to correct the imbalances in the availability of affordable healthcare facilities and to strengthen the facilities for quality medical education in the underserved states of India. All the AIIMS are autonomous bodies and are not regulated by the NMC. The study proposal was approved by Institutional Ethics Committee vide Ref. IEC/AIIMS/Kalyani/Meeting/2022/95.

Study participants

The institute was recently established in 2019, and new faculty members of different medical disciplines, dental, and nursing joined the institute. All faculty members who had never attended any FDP on the basics of health professional education technology were invited to attend the three-day FDP organized by the MEU of AIIMS Kalyani. Stakeholders (senior faculty already trained in medical education technologies holding additional administrative responsibilities) and undergraduate medical students were also included for their views.

Study type

This is a triangulation-type, mixed-methods study of four months duration, where both quantitative (quasi-experimental design) and qualitative (open-ended responses) methods were involved simultaneously for evaluating the improvement in knowledge and teaching skills.

A quasi-experimental design was adopted to evaluate the effectiveness of the intervention. The population studied was the medical faculty of AIIMS Kalyani. The intervention used was a hands-on microteaching session during FDP. A comparison was made between pre and post-intervention scores, and the outcome of the microteaching session was hypothesized as an enhancement of knowledge on the concept of the lesson plan, framing of learning objectives, and the use of appropriate teaching-learning media and effective teaching method, which is the prerequisite for developing teaching skills.

The qualitative part of the study was performed by open-ended questions used to take the views of participants and stakeholders on including microteaching sessions in the FDP.

Planning and intervention implementation

The FDP was planned with 22 topics shortlisted after reviewing the NMC document and the FDP schedule of other institutes of national importance. The average session duration was 45 minutes, and two sessions were taken as flipped classrooms (FCs). It also included three hours of mandatory microteaching sessions by following a checklist of six teaching skills, as used by Deshpande, et al., i.e., set induction, teaching planning, presentation, interaction with students, use of audio-visual (AV) aids, and summarization [8].

The microteaching was implemented in two consecutive FDPs of three days each with 20 and 17 participants (n = 37). The pre-test was conducted on 10 multiple-choice questions (MCQs) based on the concept of the lesson plan, learning objectives, and the use of appropriate teaching-learning media and other effective teaching methods. The topic of microteaching was spread across three days.

On the first day, an introductory session on microteaching was conducted through FC. On the second day, along with other topics, a detailed session on microteaching was conducted during which a handout of a checklist of six teaching skills was given to the participants. Instructions were conveyed to prepare a seven-minute topic of choice from their specialty for the microteaching session by following the teaching skills checklist. On the third day, a mandatory microteaching session in front of other participants was conducted. Four junior residents (JRs) from other departments were invited to observe the microteaching session along with members of the MEU. JRs are fresh medical graduates, and their views on how they would have preferred a particular session as students are valuable. The MEU members, JRs, and participants were divided into two groups and microteaching sessions were conducted in two parallel rooms during the same period. The classrooms were equipped with a computer, projector, whiteboard, and flip chart. The environment of the classroom was friendly and non-threatening where each participant presented a microteaching session within seven minutes. Each observer was allowed to share constructive feedback in the form of positive points about the presentation and how could it have been made more effective. Post-test was conducted on the same 10 MCQs based on teaching skills.

Feedback on strengths/opportunities and challenges/weaknesses of microteaching was taken separately from participants and stakeholders in the form of open-ended questions through Google Forms.

After two months of training, the feedback on improvement in the teaching skills of teachers was evaluated from feedback by undergraduate medical students. Online feedback forms were sent to two batches of undergraduate medical students (n = 175), 50 students in the senior and 125 in the junior batch. Information was taken on 32 faculty members who taught them in the last two months. Feedback on five faculty was not taken as they did not teach MBBS students in the past two months. They were excluded for the evaluation of Kirkpatrick’s level three. Informed consent was taken, and confidentiality was maintained at every step.

Evaluation

The evaluation of hands-on microteaching was performed to analyze its effectiveness. Because the course was evaluated in terms of learning and as it was retrospective, we used Kirkpatrick’s framework levels one, two, and three for evaluation [9].

We used participants’ feedback given for the course (level one) to decide the effectiveness of the course. Views on the strengths/opportunities and challenges/ threats from stakeholders were also evaluated. Participants’ (n = 37) and stakeholders’ (n = 7) feedback was taken via open-ended questions through Google Forms. Qualitative analysis was done using the manual content analysis method. Similar responses across the study participants were coded. Next, the codes related to similar areas were clustered together to form the categories, which were grouped under the selected themes. The Standards for Reporting Qualitative Research (SRQR) guidelines were followed while reporting [10].

The pre and post-tests were assessed manually by referring to a common answer key. The pre and end-of-course scores were compared using paired t-tests on Microsoft Excel to evaluate the gain in knowledge about teaching methods (level two).

**Figure 1:**
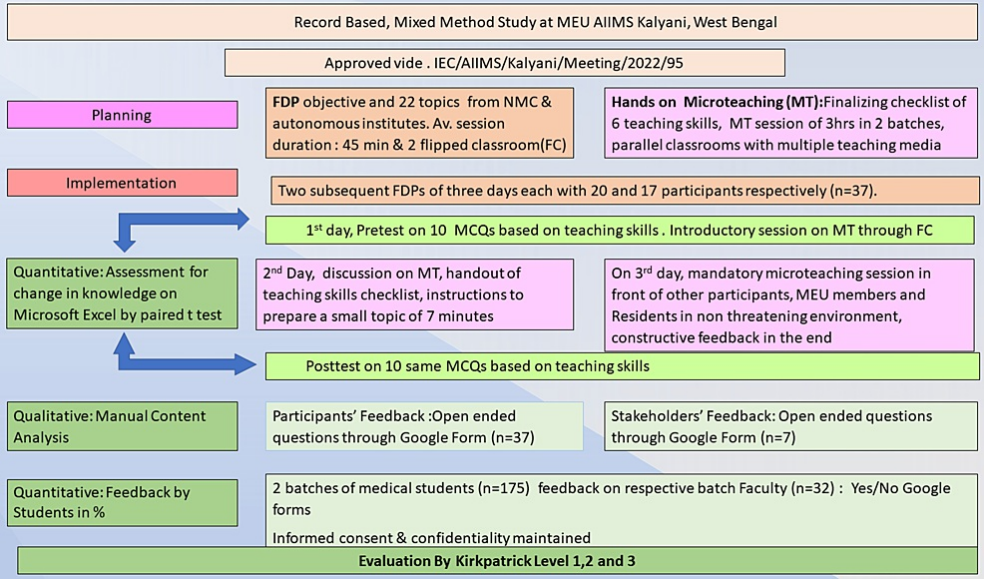
Planning, implementation, and evaluation of hands-on microteaching during the faculty development program. MEU: Medical Education Unit; AIIMS: All India Institute of Medical Sciences; NMC: National Medical Commission; MCQ: multiple-choice question

After two months of training, online feedback from MBBS students were taken to study if there was an observable improvement in the teaching skills of the teachers in the past two months (level three). The response, yes/no, was depicted in percentages. The study plan, implementation, and evaluation have been explained in Figure 1.

## Results

The results of the present study are described under three levels of the Kirkpatrick evaluation model.

Level one: reaction

The content analysis of participants’ feedback about microteaching was depicted into two themes, which are shown in Table 1, as feedback in favor of microteaching and against microteaching in FDP. The responses in favor of microteaching were that 93.5% of respondents want it to be part of FDP. Other common responses were that it builds confidence in teaching (74.2%) and helps in gaining an in-depth understanding of teaching methods (58.1%), instant feedback, hands-on experience, change in behavior, self-assessment, and time management, which were other points in favor of microteaching. Only two participants felt that selecting one small topic, time constraints, and pressure to follow guidelines are challenges in microteaching.

**Table 1:** Content analysis of participants’ feedback on microteaching.

Feedback in favor of microteaching, n (%)	Feedback against microteaching, n (%)
Should be a part of the FDP, 29 (93.5)	Challenge in selecting a small topic, 01 (3.2)
Builds confidence to teach, 23 (74.2)	Time constraints, 02 (6.4)
In-depth understanding of methods, 18 (58.1)	Pressure to follow guidelines, 1 (3.2)
Instant feedback, 08 (25.8)	
Hands-on experience, 04 (12.9)	
Changed my behavior, 03 (9.6)	
Helps in the self-assessment, 4 (12.9)	
Learned time management, 02 (6.4)	

**Figure 2:**
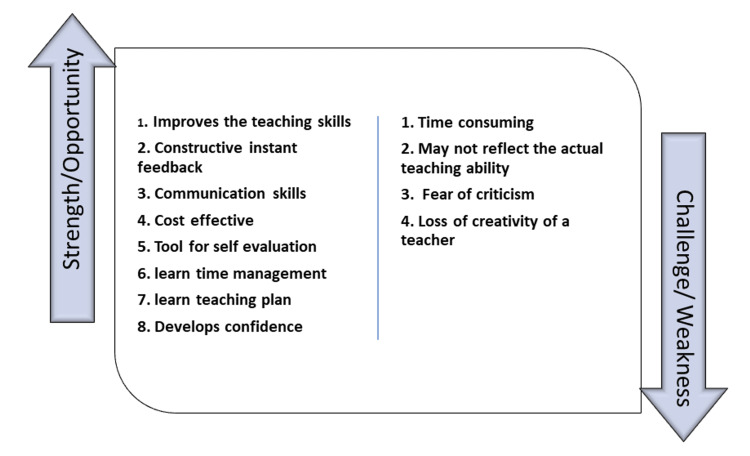
Stakeholders’ feedback on microteaching during the faculty development program.

Figure 2 shows the views of stakeholders on the strengths and challenges of the microteaching session in FDP. The response rate among stakeholders was 83.8%. They opined that the strengths and opportunities of microteaching are that it improves teaching skills, communication skills, time management, instant constructive feedback, is cost-effective, is a tool for self-evaluation, helps in learning teaching plans, and develops confidence. The challenges of microteaching are that the sessions are time-consuming, may not reflect the actual teaching ability, fear of criticism, and loss of creativity of a teacher if bound by the skill sets.

Level two: learning gained by the microteaching session in FDP

**Figure 3:**
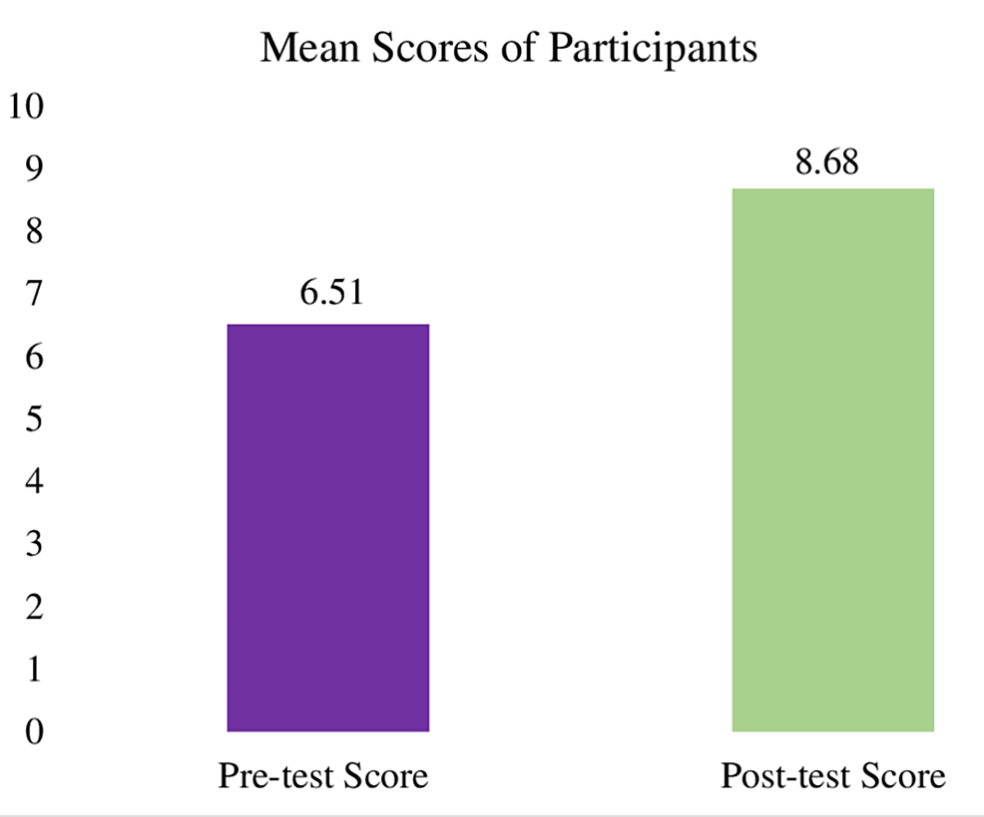
Bar diagram showing the mean scores of pre and post-test of microteaching during the faculty development program.

Figure 3 shows the mean scores obtained by the participants in pre and post-test. All 37 participants attempted the pre and post-test. The mean pre and post-test scores were 6.51 (SD = 1.63, standard error of mean = 0.27) and 8.68 (SD = 0.91, standard error of mean = 0.15), respectively (t = 7.3577, df = 36, standard error of difference = 0.294, mean difference = -2.76, 95% CI = -2.76 to -1.57, p < 0.0001).

Level three: behavior change after the microteaching session in FDP

**Figure 4:**
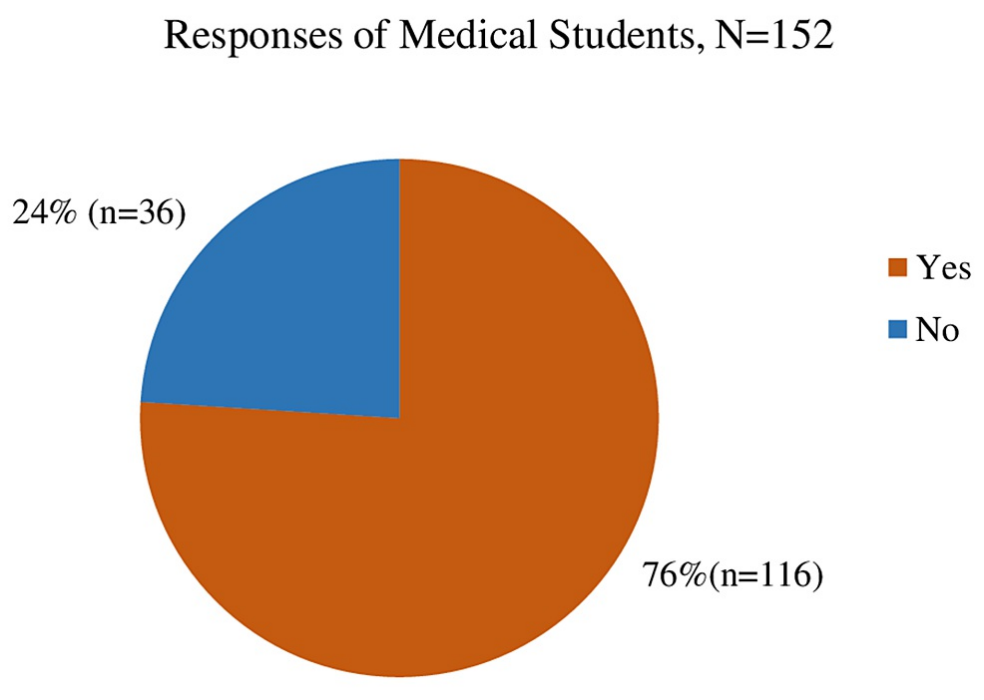
Response of undergraduate medical students regarding observable improvement in teaching skills of faculty since the past two months.

Out of 175 students, 152 responded. The response rate of undergraduate medical students was 86.8%. Figure 4 shows that the majority (76%) of students reported noticeable improvement in the teaching skills of teachers in the past two months of attending microteaching sessions.

## Discussion

Mere knowledge of pedagogy does not ensure the acquisition of teaching skills. Hands-on practice of these skills is required to build confidence and adopt them. Conducting microteaching during FDP provides a platform to acquire and practice a variety of teaching skills in front of peer groups and students/residents to get their points of view. This study was conducted to evaluate the feasibility of including hands-on microteaching in the medical FDP in India. The study used a mixed-methods approach, including both quantitative and qualitative data collection and analysis, to explore the perceptions of medical educators regarding the use of hands-on microteaching in FDPs and the change in the behavior of participants after the session.

Our findings suggest that hands-on microteaching is a feasible and effective way to improve teaching skills among medical educators in India. The quantitative data showed a significant improvement in the teaching skills of participants after they underwent hands-on microteaching sessions. This is consistent with previous research that has shown microteaching to be an effective teaching technique for improving the teaching skills of educators in various fields [3-5].

For the microteaching session, the presenter was given a checklist of the six core teaching skills and their components for consideration while preparing the presentation similar to a study reported by Deshpande et al. [8]. Studies have reported that microteaching improves the fluency, speaking ability, and confidence of the teacher [11,12]. The literature has endorsed the use of these core skill sets to improve the quality of teaching [11-15]. The majority of undergraduate medical students (76%) felt a noticeable improvement in the teaching skills of the teachers in the past two months after attending the microteaching sessions.

Our results are consistent with several studies that show that microteaching is a very effective method for improving teaching skills, communication, and time management skills. Moreover, it enhances confidence, better lesson plans, appropriate use of AV aids, students’ engagement, and active teaching-learning [3,8,11-18]. The qualitative data also provided valuable insights into the perceptions of medical educators regarding the use of hands-on microteaching in FDPs. Participants reported that the hands-on approach helped them to better understand and apply teaching concepts and provided them with opportunities to receive feedback from their peers and mentors. This is consistent with literature that has shown the value of feedback in improving teaching skills [4,5,13,14]. One of the main challenges in microteaching as reported by participants is time-consuming sessions, also reported by Dayanidhi et al. [11]. In this study, time was managed by an introduction to microteaching by FC on day one, and conducting the hands-on sessions in parallel classrooms on day three. Stakeholders consider that fear of criticism during the session is another weakness of microteaching. It is very important to provide feedback in a friendly and non-threatening environment in adult learning. In our study, the participants enjoyed their presentations and learned the skills by observing feedback for other participants as well. No participant mentioned criticism or humiliation in anonymous feedback. Some participants and stakeholders opine that pressure to follow guidelines may lead to a loss of creativity in the teacher. It is important to make the session effective, and in short seven minutes, many components of core skills may not be followed.

Studies have reported that microteaching should be video recorded and provided to participants as it is useful in self-introspection and reflection on one’s performance. Participants felt it was very important to provide them with recordings of their presentations to enable them to get a 360-degree assessment of their teaching which serves as a very strong trigger for behavioral change [19-21]. The NMC has mandated a skill and simulation lab equipped with video recording in each medical college, which can also be utilized for the skill development of teachers by video recording microteaching sessions.

Limitations of the study

It is a challenge to ensure the homogeneity of the study participants as the teaching faculty have varying proficiency levels and prior teaching experiences. Longitudinal follow-up is required to evaluate level four of Kirkpatrick’s model to study the effect of microteaching on the outcome, that is, the performance of undergraduate medical students during their assessment.

## Conclusions

This mixed-methods study evaluated the feasibility of including hands-on microteaching in the medical FDP in India. The study found that hands-on microteaching is a feasible and effective tool for enhancing the teaching skills of medical educators. Our study contributes to the existing literature on FDPs by demonstrating the potential of hands-on microteaching as a cost-effective and scalable approach for improving the teaching skills of medical educators in India. All medical institutions conduct FDPs that consume organizational time and resources. A little adjustment in the given schedule to include hands-on microteaching sessions in a non-threatening environment will lead to better teaching skills with confidence and behavior change making lessons more interesting and effective.

We recommend that NMC India considers incorporating hands-on microteaching into the schedule of FDPs, as it can help bridge the gap between theoretical knowledge and practical application of teaching skills.
